# Modulating the Inflammatory Response to Wounds and Cancer Through Infection

**DOI:** 10.3389/fcell.2021.676193

**Published:** 2021-04-30

**Authors:** Paco López-Cuevas, Stephen J. Cross, Paul Martin

**Affiliations:** ^1^School of Biochemistry, University of Bristol, Bristol, United Kingdom; ^2^Wolfson Bioimaging Facility, University of Bristol, Bristol, United Kingdom; ^3^School of Physiology, Pharmacology and Neuroscience, University of Bristol, Bristol, United Kingdom

**Keywords:** zebrafish, cancer, wound healing, infection, Coley’s toxins, inflammation, neutrophil, macrophage

## Abstract

The zebrafish (*Danio rerio*) has recently emerged as an excellent model to study cancer biology and the tumour microenvironment, including the early inflammatory response to both wounding and early cancer growth. Here, we use high-resolution confocal imaging of translucent zebrafish larvae, with novel automated tracking and cell:cell interaction software, to investigate how innate immune cells behave and interact with repairing wounds and early cancer (pre-neoplastic) cells expressing a mutant active human oncogene (HRASG12V). We show that bacterial infections, delivered either systemically or locally, induce a change in the number and behaviour of neutrophils and macrophages recruited to acute wounds and to pre-neoplastic cells, and that infection can modify cellular interactions in ways that lead to a significant delay in wound healing and a reduction in the number of pre-neoplastic cells. Besides offering insights as to how Coley’s toxins and other cancer bacteriotherapies may function to reduce cancer burden, our study also highlights novel software tools that can be easily adapted to investigate cellular behaviours and interactions in other zebrafish models.

## Introduction

There are many cell and molecular parallels between wound healing and cancer, and it has often been said that tumours behave somewhat like wounds that fail to heal (Dvorak, 1986; Schäfer and Werner, 2008; MacCarthy-Morrogh and Martin, 2020). Both wounds and cancer activate an inflammatory response and both tissue insults trigger release of several of the key “damage” attractants, including H_2_O_2_, HMGB1 and chemokines that bind CXCR2, which drive innate immune cell recruitment (Niethammer et al., 2009; Feng et al., 2010; Moreira et al., 2010; Willenborg et al., 2012; de Oliveira et al., 2013; Bald et al., 2014; Freisinger and Huttenlocher, 2014; Coombs et al., 2019; Zhou et al., 2020). Similarly, some of the downstream consequences of localised inflammation, including matrix deposition and angiogenesis, are common to cancer and wound healing also (MacCarthy-Morrogh and Martin, 2020). For both cancer and wound healing, the inflammatory response can be considerably altered by the local microbiome and by local, or systemic infection (Holder et al., 1997; Garrett, 2015; Dzutsev et al., 2017; Williams et al., 2017; Helmink et al., 2019; Janney et al., 2020). Serendipitous findings extending back to those of Coley in the early 1900s have suggested that infection can enhance, or prime, the host immune response to better recognise and eradicate cancers (Coley, 1910; Felgner et al., 2016). During tissue repair, there is evidence that some aspects of the wound inflammatory response may be activated by exposure to microbial antigens (Holder et al., 1997; Crompton et al., 2016; Miskolci et al., 2019; Schild et al., 2020), although if infection becomes overwhelming, this can lead to a chronic non-healing wound (Caldwell, 2020). Whilst the longer term consequences of infection on cancer and wound healing have been partially explored, rather little is known about how infections alter the behaviour of innate immune cells, to influence these consequences, for example their velocity and migratory persistence towards, and in the vicinity of the tissue insult, and how this in turn impacts on wound or cancer pathology. Here we take advantage of the genetic tractability and translucency of zebrafish larval tissue to perform high resolution imaging (with and without infection), of immune cell interactions with wounds, and during cancer initiation, which we analyse and quantify using a bespoke automated cell tracking and cell:cell interaction workflow.

## Results and Discussion

### Inflammatory Cell Behaviour Is Similar in Response to Wounds and the Presence of Pre-neoplastic Cells

The recruitment of innate immune cells to early pre-neoplastic clones and to wounds can profoundly influence cancer and repair outcomes (Feng et al., 2010; Antonio et al., 2015; Gurevich et al., 2018; Loynes et al., 2018). Detailed studies of inflammatory cell interactions and behaviours once having reached the vicinity of their target site have been hindered by a lack of suitable bespoke automated tracking software. We have developed novel algorithms to compare macrophage and neutrophil recruitment behaviours in response to growing pre-neoplastic clones vs. acute injury.

For a cancer model, we took advantage of a previously published zebrafish line, Tg(*kita:HRASG12V-GFP*) (Feng et al., 2010; Santoriello et al., 2010), where the kita promoter drives gene expression of a mutant human oncogene, HRASG12V, tagged with GFP, in melanoblasts and mucus-secreting goblet cells of the zebrafish larval skin. We have focused our studies on goblet cells in this cancer model because of their clarity for imaging. These HRASG12V expressing cells multiply unlike their equivalents in control larvae which express only a fluorescent marker and remain as single cells; from here on in we term the HRASG12V expressing clones as pre-neoplastic cells. For acute injury we made needle stick local wounds in the flanks of larvae.

**FIGURE 1:**
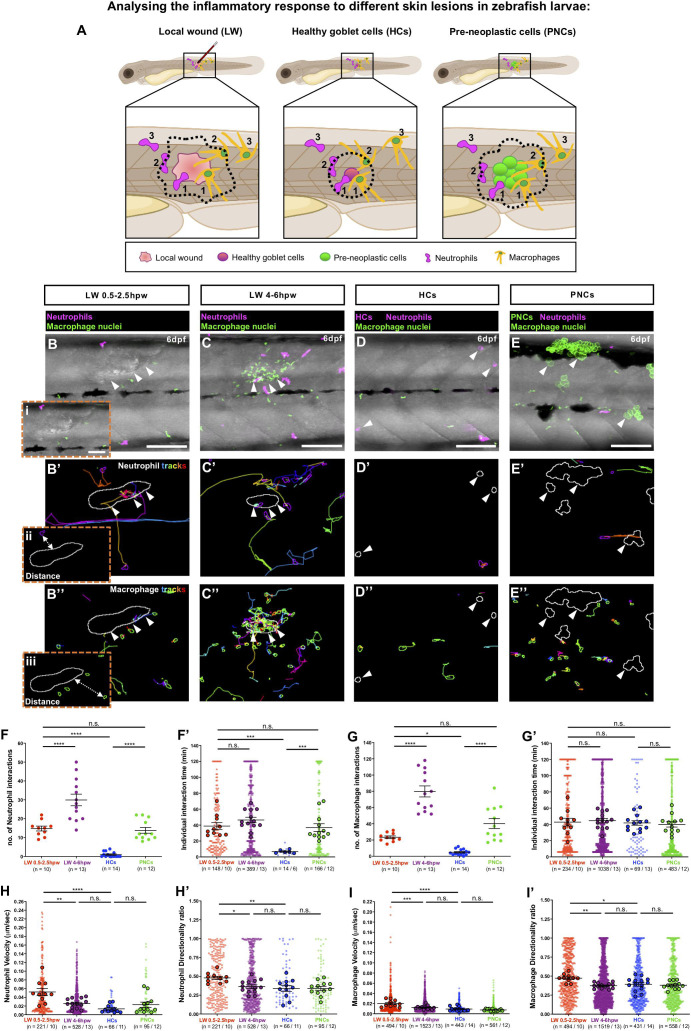
Analysing the inflammatory response to different skin lesions in zebrafish larvae. **(A)** Schematic to illustrate our cancer-wound comparison studies showing the region (black box) to be imaged in **(B–E)**, for neutrophils (magenta) and macrophages (orange) as they respond to the different skin lesions: local wound (LW), healthy RFP-expressing goblet cells (HCs) or pre-neoplastic GFP-expressing goblet cells (PNCs); dotted lines indicate the “close proximity” zone from margins of each lesion type; 1 and 2 indicate immune cells in contact with the lesion or within “close proximity”, respectively, and 3 indicates immune cells outside this zone. **(B–E)** Multi-channel confocal movie frames of 6 dpf flank wounded Tg(*lyz:DsRed;mpeg1:nls-Clover*) **(B,C)**, unwounded control Tg(*kita:mCherry; lyz:DsRed;mpeg1:nls-Clover*) **(D)** or cancerous Tg(*kita:HRASG12V-GFP;lyz:DsRed;mpeg1:nls-Clover*) **(E)** larvae prior to analysis of neutrophil (magenta) or macrophage (green nuclei) behaviour and their interactions with the respective skin lesions (white arrowheads). **(B’–E’,B”–E”)** Post-software images of the same larvae showing neutrophils (magenta) **(B’–E’)** or macrophage nuclei (green) **(B”–E”)** and their tracks in the vicinity of the lesion (white lines indicate lesion margins). **(i–iii)** Higher magnification views from **(B,B’,B”)** showing the “cell-to-cell” distance between neutrophil cytoplasmic (ii) or macrophage nuclear (iii) margins to lesion margins. **(F,G,F’,G’)** Graphs showing number and duration of neutrophil-lesion **(F,F’)** and macrophage-lesion **(G,G’)** interactions. **(H,I,H’,I’)** Graphs showing velocity and directionality ratio of neutrophils **(H,H’)** and macrophages **(I,I’)** quantified at the lesion site. Scale bars = 100 μm in **(B–E)**, 50 μm in **(Bi)**.

We first compared the behaviour of innate immune cells responding to multiple clones of pre-neoplastic cells vs. wounded tissue, by live imaging neutrophils expressing cytoplasmic RFP and macrophages expressing nuclear GFP in Tg(*kita:HRASG12V-GFP;lyz:DsRed;mpeg1:nls-Clover*) cancerous larvae at 6 days post-fertilisation (dpf) (Hall et al., 2007; Bernut et al., 2019), and wounded Tg(*lyz:DsRed;mpeg1:nls-Clover*) larvae at the same developmental stage. Equivalent regions of skin in cancerous fish with pre-neoplastic clones, and in control larvae with single goblet cells fluorescently tagged with RFP, were imaged for 2 h, and these movies were compared to 2 h movies collected from wound lesions made in larvae with fluorescently labelled immune cells but no cancer burden at either 0.5 or 4 h post-wounding (hpw). Using a bespoke workflow enabling automated analysis we are able to quantify interactions between immune cells and lesions (wounds or pre-neoplastic cells) over time (Figure 1A and Supplementary Figure 1). This algorithm was designed to accurately detect and outline the plasma membrane of neutrophils, and the nuclei of macrophages, as well as the margins of both pre-neoplastic cancer clones and wounds, enabling dynamic “cell-to-cell” distance measurements between immune cells and the corresponding lesions to be gathered from large movie datasets (Figures 1Bi-iii and Supplementary Movie 1).

Using this software we quantified the number of immune cell-lesion interactions [classified as direct contacts or those within close proximity (<20 μm or <50 μm, neutrophil margin and macrophage nuclei, respectively)], and their duration (Figure 1A), in order to distinguish whether downstream consequences might be dependent on direct cell-lesion contacts, or rather, mediated by diffusible signals (e.g., growth factors or cancer cell killing molecules).

Very few neutrophil-goblet cell interactions were detected in unwounded control larvae (Figures 1D,F,F’ and Supplementary Movie 1). However, as previously described (Feng et al., 2010), if these cells express mutant HRASG12V, neutrophils are recruited to and interact with the growing pre-neoplastic clones (Figures 1E,F,F’ and Supplementary Movie 1). We see a similar level of recruitment, retention and interaction of neutrophils with larval local flank wounds at early timepoints (0.5–2.5 hpw) post-wounding (Figures 1B,F,F’ and Supplementary Movie 1). At later timepoints (4–6 hpw), the number of neutrophil-wound interactions increased considerably (Figures 1C,F and Supplementary Movie 1).

Numbers of macrophage interactions with both pre-neoplastic clones and local flank wounds are also higher than for control fish, with a progressive increase in the number of interactions observed at later timepoints post-wounding (Figures 1B–E,G and Supplementary Movie 1). Individual macrophage-lesion interaction time was similar for fish with pre-neoplastic clones and local flank wounds (Figure 1G’ and Supplementary Movie 1).

As well as counting numbers of immune cell interactions and measuring their interaction times with these various lesions, we wanted to determine whether their migratory behaviour, as gleaned from their tracks, had been significantly altered. Both neutrophil and macrophage velocity and directionality towards local flank wounds increase as cells are first being recruited, after wounding (from 0.5 to 2.5 hpw), but this reverted back to control levels by 4 h post-wounding (Figures 1H,I,B’–D’,H’,I’,B”–D” and Supplementary Movie 1). By contrast, despite clear recruitment of individual immune cells to pre-neoplastic cells, we see no overall increase in neutrophil or macrophage velocity, or directionality in the vicinity of these clones (Figures 1H,I,E’,H’,I’,E” and Supplementary Movie 1), indicating that recruitment of immune cells to pre-neoplastic clones was less synchronised than to wounds and thus hidden in our analyses of these multiple independently acting clones.

### Infection Alters the Wound Inflammatory Response

**FIGURE 2:**
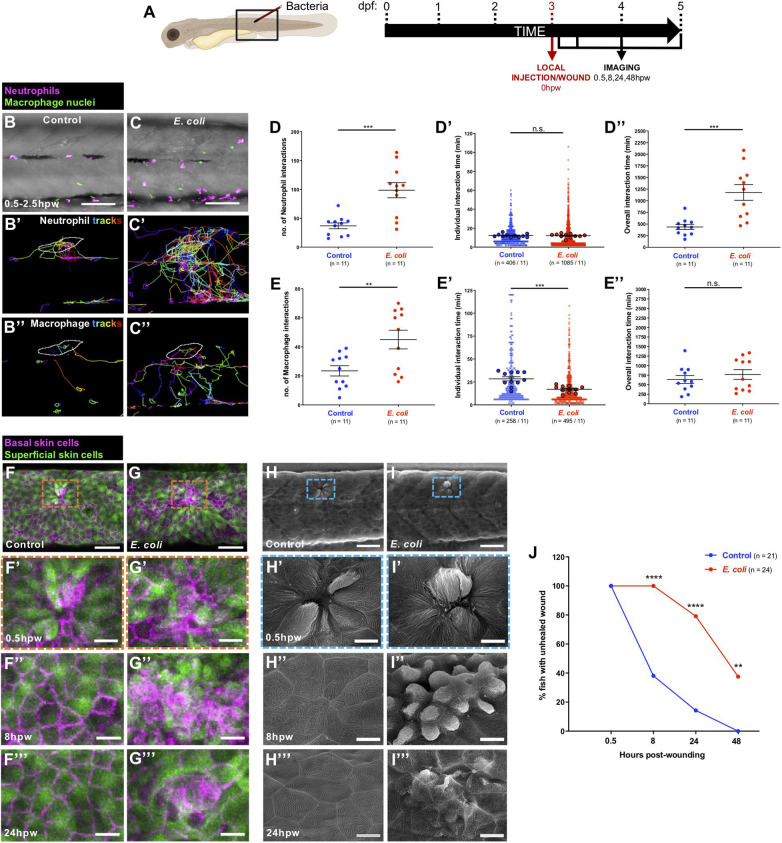
Alterations to the wound inflammatory response upon *E. coli* infection and consequences for tissue repair. **(A)** Schematic of the experimental timeline for locally infected flank wound studies showing the region (black box) to be imaged in **(B,C,F–I)**. **(B,C)** Multi-channel confocal movie frames of flank wounded Tg(*lyz:DsRed;mpeg1:nls-Clover*) larvae at 0.5 hpw after local injection of control media **(B)** or *E. coli* **(C)**, and prior to analysis of neutrophil (magenta) or macrophage (green nuclei) behaviour and their interactions with the wound. **(B’,C’,B”,C”)** Post-software images of the same larvae showing neutrophils (magenta) **(B’,C’)** or macrophage nuclei (green) **(B”,C”)** and their tracks towards the wound (white lines indicate wound margins). **(D,E,D’,E’,D”,E”)** Graphs showing number and individual/overall duration of neutrophil-wound **(D,D’,D”)** and macrophage-wound **(E,E’,E”)** interactions. **(F,G)** Multi-channel confocal images of flank wounded Tg(*krt4:GFP;krt19:tdTomato-CAAX*) larvae showing the disposition of the larval zebrafish skin superficial (green) and basal (magenta) cell layers at 0.5 hpw in control **(F)** or *E. coli*-injected fish **(G)**. **(H,I)** Scanning electron micrographs of flank wounded larvae at 0.5 hpw after local injection of control media (H) or *E. coli* **(I)**. **(F’–I’,F”–I”,F”’–I”’)** Higher magnification views of wound regions from **(F–I)** at 0.5 hpw **(F’–I’)**, 8 hpw **(F”–I”)**, and 24 hpw **(F”’–I”’)**. **(J)** Graph showing rate of healing in infected vs. uninfected wounds. Scale bars = 100 μm in **(B,C)**, 75 μm in **(F,G)**, 50 μm in **(H,I)**, 20 μm in **(F’,G’,F”,G”,F”’,G”’)**, 10 μm in **(H’,I’,H”,I”,H”’,I”’)**.

Next, we investigated whether bacterial infection alters the inflammatory response to wounding. We selected the gram-negative bacterium *E. coli*, which is non-pathogenic, but triggers an inflammatory response when injected into zebrafish larvae (Sieger et al., 2009; Colucci-Guyon et al., 2011; Hou et al., 2016). To address how neutrophils and macrophages respond to an infected wound, Tg(*lyz:DsRed;mpeg1:nls-Clover*) larvae were locally injected with *E. coli* (or control media) into a somite at 3 dpf, and larvae were imaged from 0.5 to 2.5 hpw (Figures 2A–C and Supplementary Movie 2). Infection leads to an increase in numbers of both neutrophil and macrophage interactions with the wound lesion. For neutrophils this leads to an increased total interaction time in infected vs. uninfected wounds. However, for macrophages, individual interactions become shorter in duration so that the total duration of macrophage interactions is not significantly altered by infection (Figures 2B–E,D’,E’,D”,E” and Supplementary Movie 2). Infection also alters other behavioural aspects of immune cells, in the vicinity of wounds, increasing their migration velocity although not their directionality (Figures 2B’,C’,B”,C”, Supplementary Figures 2A,B,A’,B’, and Supplementary Movie 2). Our time-lapse movies also show that infected wounds induce robust neutrophil swarming (Supplementary Movie 2), in agreement with data reported by others in response to different infectious stimuli (Huang and Niethammer, 2018; Poplimont et al., 2020).

Because infection clearly impacts the wound inflammatory response, we questioned how this might alter wound healing *per se*. To study this, we used confocal and scanning electron microscopy to image wounded Tg(*krt4:GFP;krt19:tdTomato-CAAX*) larvae in which superficial and basal epidermal skin cells are labelled in green and red, respectively (van den Berg et al., 2019) (Figures 2F–I). We observed that infection significantly delays wound healing of needle stick local wounds. Uninfected wounds heal very rapidly within the first hours post-wounding (Gault et al., 2014; Morris et al., 2018), with 60% of wounded fish showing complete re-epithelialisation by 8 hpw (Figures 2F”,H”,J). However, none of the infected wounds are healed at this early timepoint (Figures 2G”,I”,J), and while 100% of control wounds have completely healed by 48 hpw, 40% of infected wounds still remain unhealed and disorganised (Figure 2J), although at later timepoints they heal completely (data not shown). Infection is a known risk factor for impaired healing of wounds (Holder et al., 1997; Crompton et al., 2016; Miskolci et al., 2019) and our data suggest that a key link might be, at least in part, an altered wound inflammatory response, and that inflammatory cell behaviour might serve as a prognostic indicator for wound healing status.

### Infection Triggers an Increase in Leukocyte Numbers and Velocity in Otherwise Healthy Skin

Prior to our infection studies in cancerous larvae, we investigated how bacterial infection modulates the number and behaviour of immune cells in otherwise healthy zebrafish skin. Tg(*lyz:DsRed;mpeg1:nls-Clover*) larvae were either systemically injected with *E. coli* (or control media) at 2 dpf, or locally injected at 3 dpf, and the resulting behaviour of innate immune cells at the local injection site was quantified at 2 days post-injection (dpi) (Supplementary Figures 3A,F). Systemic *E. coli* infection triggers an increase in both numbers of macrophages and their velocity in skin, but does not appear to alter neutrophil numbers or their behaviour (Supplementary Figures 3B–E,B’–E’,B”–E” and Supplementary Movie 3). Local *E. coli* infection leads to increased numbers of both macrophages and neutrophils in the skin, and analysis of their tracks shows that both lineages exhibit increased velocity, with neutrophils also showing increased directionality, as if to an acute wound (Supplementary Figures 3G–J,G’–J’,G”–J” and Supplementary Movie 4).

### Systemic and Local *E. coli* Infections Both Alter the Kinetics of Interactions Between Inflammatory Cells and Pre-neoplastic Cells

**FIGURE 3:**
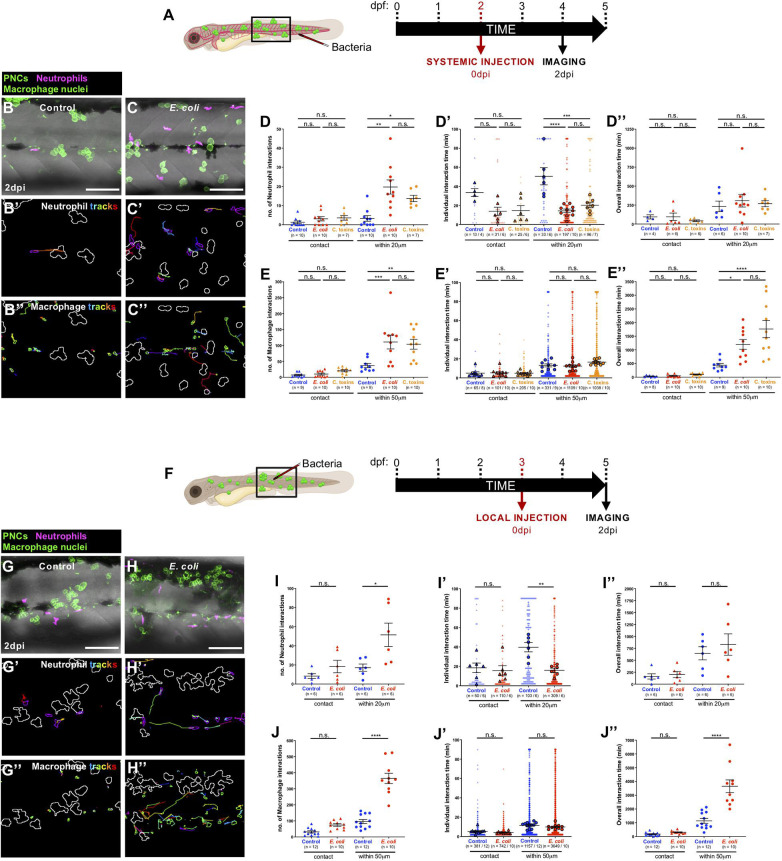
Altered cancer inflammatory response upon *E. coli* or Coley’s toxins infection. **(A)** Schematic of the experimental timeline for systemically infected cancer studies showing the region (black box) to be imaged in **(B,C)**. **(B,C)** Multi-channel confocal movie frames of the flank of cancerous Tg(*kita:HRASG12V-GFP;lyz:DsRed;mpeg1:nls-Clover*) larvae at 2 dpi after systemic injection of control media **(B)** or *E. coli* **(C)**, and prior to analysis of neutrophil (magenta) or macrophage (small green nuclei) behaviour and their interactions with pre-neoplastic cells (large green cells). **(B’,C’,B”,C”)** Post-software images of the same larvae showing neutrophils (magenta) **(B’,C’)** or macrophage nuclei (green) **(B”,C”)** and their tracks in the vicinity of pre-neoplastic clones (white lines indicate clonal margins). **(D,E,D’,E’,D”,E”)**. Graphs showing number and individual/overall duration of neutrophil-cancer **(D,D’,D”)** and macrophage-cancer **(E,E’,E”)** interactions. **(F)** Schematic of the experimental timeline for locally infected cancer studies showing the region (black box) to be imaged in **(G,H)**. **(G–J,G’–J’,G”–J”)** The same analysis was carried out for local infection as previously described for the systemic infection experiments. Scale bars = 100 μm.

Next, we examined how innate immune cells respond to pre-neoplastic cells in the skin, in the presence of a local or systemic *E. coli* infection, due to the potential clinical relevance of both infection strategies in the treatment of local primary cancers or dispersed metastatic cancers, respectively. Therefore, Tg(*kita:HRASG12V-GFP;lyz:DsRed;mpeg1:nls-Clover*) cancerous larvae were injected with *E. coli* (or control media), as previously described (Figures 3A,F). Both systemic and local infection experiments showed that immune cells are recruited in higher numbers to the cancerous skin (Supplementary Figures 4A–D) and that the number of occasions when leukocytes came within close proximity of a clone of pre-neoplastic cells is significantly increased in *E. coli* injected-fish compared with uninfected fish at 2 dpi (Figures 3B–E,G–J and Supplementary Movies 5, 6). However, for neutrophils, each of these interaction times are shorter so that total duration of neutrophil interactions is not significantly altered by infection (Figures 3D’,I’,D”,I”). By contrast, individual macrophage:pre-neoplastic cell interactions are of similar duration time, leading to an overall increase in interaction time in the infected fish (Figures 3E’,J’,E”,J”). Both neutrophils and macrophages exhibit an increased velocity, but no alteration in their directionality, when cancerous larvae are systemically or locally infected with *E. coli* compared with uninfected fish at 2 dpi (Figures 3B’,C’,G’,H’,B”,C”,G”,H”, Supplementary Figures 4A’–D’,A”–D”, and Supplementary Movies 5, 6).

Our automated cell tracking algorithm allows us to distinguish between direct contacts and close interactions between neutrophils and pre-neoplastic cancer clones. When we quantify number and duration of neutrophil:pre-neoplastic cell direct contacts we see no significant difference between infected and control larvae, in both systemic and local experiments, and similar is true for macrophage:pre-neoplastic cell direct contacts (Figures 3D,E,I,J,D’,E’,I’,J’,D”,E”,I”,J”) suggesting that any immune cell influence on cancer progression is not entirely dependent on direct cell:cell contacts, and rather is likely to be, at least in part, mediated by diffusible signals. Previous live imaging studies in zebrafish have indicated that direct contacts occur and can even lead to phagocytosis of pre-neoplastic cells (Feng et al., 2010; Chia et al., 2018), or to “donation” of cytoplasmatic material to the cancer cells (Roh-Johnson et al., 2017). However, murine studies have shown that infection-mediated tumour growth suppression is mediated by pro-inflammatory cytokines secreted by tumour-associated macrophages (Kim et al., 2015).

### Coley’s Toxins Mimics *E. coli*-Triggered Immune Cell Responses to Pre-neoplastic Cells

As previously described, a combination of heat-killed bacteria (*S. pyogenes* and *S. marcescens*), termed Coley’s toxins, has previously been shown to trigger cancer regression when administered to cancer patients (Coley, 1910; Felgner et al., 2016). This cancer “dissolving” effect was thought to be mediated by the activation of the host immune system following toxin treatment (Wiemann and Starnes, 1994). Therefore, we wondered whether Coley’s toxins might reproduce the behavioural effects of immune cells observed when *E. coli* was systemically injected in cancerous larvae. Indeed, we find that both neutrophils and macrophages alter their numbers and behaviour in the cancerous skin, and their interactions with pre-neoplastic cells upon systemic injection of Coley’s toxins, in ways that mimic our *E. coli* data (Figures 3D,E,D’,E’,D”,E” and Supplementary Figures 4A,B,A’,B’,A”,B”).

### A Longer Term Consequence of Altered Immune Cell Behaviours Post-infection Is a Reduction in Numbers of Pre-neoplastic Cells

**FIGURE 4:**
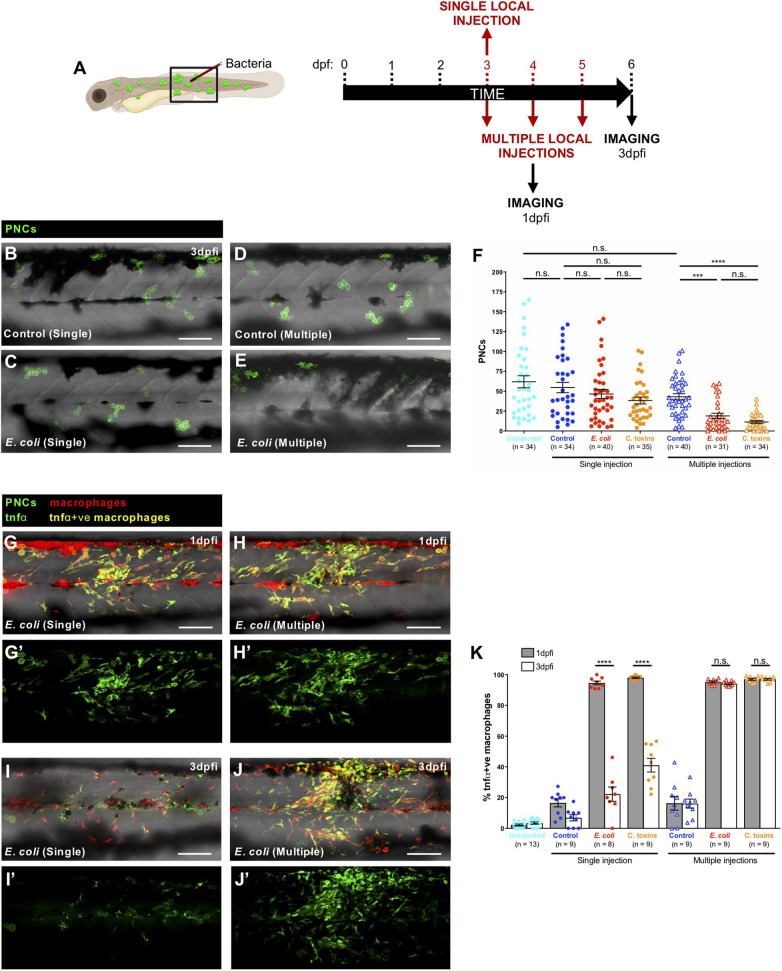
Prolonged pro-inflammatory response and reduction in cancer cell numbers upon consecutive local injections of *E. coli* or Coley’s toxins. **(A)** Schematic of the experimental timeline for single/multiple locally infected cancer studies showing the region (black box) to be imaged in **(B–E,G–J)**. **(B–E)** Multi-channel confocal images of the flank of cancerous Tg(*kita:HRASG12V-GFP*) larvae showing pre-neoplastic cells (green) at 3 dpfi after single vs. multiple local injections of control media **(B,D)** or *E. coli* **(C,E)**. **(F)** Graph showing number of pre-neoplastic cells following each treatment. **(G–J)** Multi-channel confocal images of the flank of cancerous Tg(*kita:HRASG12V-GFP;mpeg1:mCherry;tnf*α*:GFP*) showing pre-neoplastic cells (green) and tnfα-positive macrophages (yellow) (tnfα-negative macrophages are red) at 1 dpfi **(G,H)** or 3 dpfi **(I,J)** after single vs. multiple local injections of *E. coli*. **(G’–J’)** Single-channel confocal images of the same larvae showing GFP-expressing cells (pre-neoplastic cells and tnfα-positive cells). **(K)** Graph showing the percentage of tnfα-positive macrophages at two timepoints for all of the injection regimes. Scale bars = 100 μm.

Since administration of live bacteria or Coley’s toxins to larvae with pre-neoplastic lesions clearly alters the behaviour of immune cells and their interactions with these pre-neoplastic cells, we investigated what might be the longer term consequences of these changes. A single systemic, or local injection of *E. coli* or Coley’s toxins at 3 dpf, results in no long term effect in numbers of pre-neoplastic cells (Figures 4A–C,F), but when cancerous larvae were given consecutive local injections of *E. coli* or Coley’s toxins at 3, 4, and 5 dpf, we observe that the number of pre-neoplastic cells is significantly reduced in 6 dpf larvae compared with fish multiply injected with control media (Figures 4A,D–F).

As a first step in characterising this infection-triggered blocking influence on immune cells we decided to investigate how multiple infections might alter leukocyte phenotype in ways that differ from a single infection timepoint. Tumour necrosis factor α (tnfα) expression is a useful proxy for the pro-inflammatory or M1 macrophage phenotype (Nguyen-Chi et al., 2015; Gurevich et al., 2018), and has been linked to cancer cell killing (Laster et al., 1988; Balkwill, 2009; Kim et al., 2015; Póvoa et al., 2021). We combined a zebrafish tnfα reporter line, Tg(*tnf*α*:GFP*) (Marjoram et al., 2015), with Tg(*kita:HRASG12V-GFP;mpeg1:mCherry*) cancerous fish, to reveal macrophages with a pro-inflammatory (tnfα-positive) phenotype in the vicinity of pre-neoplastic cells. Our data indicate that single injections of *E. coli* or Coley’s toxins lead only to transient tnfα expression by skin macrophages but daily injections lead to chronic tnfα expression maintained from 1 to 3 days post-first injection (dpfi) (Figures 4G–K,G’–J’). We speculate that this prolonged pro-inflammatory state exhibited by macrophages after multiple local infections may prompt the reduced pre-neoplastic cell numbers that we observe, although experiments transiently inhibiting these chronically activated tnfα-positive macrophages are needed to definitively confirm this. If proven, this would suggest a threshold response which we have seen also when investigating how wounding impacts on cancer cell growth, where only wounds above a threshold size, and triggering a sufficiently large inflammatory response, impact on clonal growth (Antonio et al., 2015).

Our study provides a new approach for quantifying how infection may alter the inflammatory response to a wound and to cancer cells *in vivo*, and how this might trigger immune cells to inhibit cancer growth or encourage cancer killing (Supplementary Table 1). Clearly, in the longer term it will be more useful to develop therapies that avoid delivery of infective particles. In this regard, a recent study showing how bacterial antigens can be mimicked by specific Toll-Like Receptor 2 (TLR2) agonists to switch macrophages into an anti-tumour phenotype indicates that such approaches may lead us towards therapies for the clinic (Feng et al., 2019).

## Materials and Methods

### Zebrafish Lines and Maintenance

Husbandry of adult zebrafish (*Danio rerio*) was performed as previously described (Westerfield, 2007). We used transgenic lines including: Tg(*kita:Gal4;UAS:mCherry;UAS:HRASG12V-GFP*) (Feng et al., 2010; Santoriello et al., 2010) which were in-crossed to obtain siblings either cancerous Tg(*kita:HRASG12V-GFP*), or control Tg(*kita:mCherry*); Tg(*lyz:DsRed*) (Hall et al., 2007); Tg(*mpeg1:nls-Clover*) (Bernut et al., 2019); Tg(*mpeg1:mCherry*) (Ellett et al., 2011); Tg(*krt4:GFP;krt19:tdTomato-CAAX*) (van den Berg et al., 2019); Tg(*tnf*α*:GFP*) (Marjoram et al., 2015). Animal experiments were ethically approved by the University of Bristol Animal Welfare and Ethical Review Body (AWERB) and conducted in accordance with the UK Home Office regulations.

### Bacterial Infection

*E. coli* infection experiments were performed using *E. coli* (BL21), expressing red fluorescent protein DsRed (kindly provided by Will Wood, University of Edinburgh) or blue fluorescent protein BFP (Thermo Fisher Scientific). Injection inoculum was prepared from an overnight LB culture in the log-phase of growth resuspended in 2% polyvinylpyrrolidone40 (PVP40) solution (Sigma) and phenol red (Sigma). For systemic infection, zebrafish larvae were anaesthetised at 2 days post-fertilisation (dpf) by immersion in 0.16 mg/mL Tricaine (Sigma) and injected with 2 nL containing 400–500 colony forming units (CFUs) of *E. coli* into the caudal vein, as previously described (Takaki et al., 2013). This bacterial concentration was selected as sub-lethal from our own titration experiments, and in accordance with previous studies (Colucci-Guyon et al., 2011). For localised infection, 1.5 nL containing 1,500–1,600 CFUs of *E. coli* were injected subcutaneously in the somite above the cloaca of anaesthetised 3 dpf zebrafish larvae, as previously described (Benard et al., 2012).

Coley’s toxins infection experiments were performed using Coley Fluid (MBVax Bioscience, Canada) which contains a combination of heat-killed *S. pyogenes* and *S. marcescens* equivalent to the preparation used by W. Coley in the past (Wiemann and Starnes, 1994), resuspended in 2% PVP40 solution. Zebrafish larvae were injected with 2 nL of this preparation for systemic infection or 1.5 nL for localised infection.

For repetitive local infections experiments, *E. coli* or Coley’s toxins were locally injected in the same somite (above the cloaca) on consecutive days (3, 4, and 5 dpf).

### Wounding Protocols

For localised wounding experiments, zebrafish larvae were anaesthetised in 0.16 mg/mL Tricaine prior to wounding their flanks in the somite directly adjacent the cloaca using a glass needle, following the protocol previously described (Gurevich et al., 2018). Larvae were harvested at appropriate times post-wounding and analysed by confocal and scanning electron microscopy (see below). Timepoints for analysis of immune cell-wound interactions were restricted to up to 6 h post-wounding to capture the early inflammatory response before the neutrophils leave the wounded area and a healthy skin wound entirely seals.

### Scanning Electron Microscopy

Zebrafish larvae were anaesthetised and fixed in primary fix (2.5% glutaraldehyde, 4% paraformaldehyde, 0.1 M sodium cacodylate) at 4°C overnight. These samples were washed in 0.1 M sodium cacodylate (3 × 10 min) and then transferred to 1% osmium tetroxide, at room temperature for 2 h. After fixation, samples were rinsed in 0.1 M sodium cacodylate before serial dehydration in EtOH and critically point dried and sputter coated with Gold/Palladium (Au/Pd) prior to imaging with a FEI Quanta 200 FEG scanning electron microscope.

### Confocal Imaging

Anaesthetised zebrafish larvae were mounted in 1% low-melting point agarose (Sigma) in a glass-bottomed dish, filled with Danieau’s solution with 0.1 mg/mL of Tricaine anaesthetic. Images were collected using a Leica TCS SP8 AOBS confocal laser scanning microscope attached to a Leica DMi8 inverted epifluorescence microscope with a 20× glycerol lens, maintained at 28°C. Movies were recorded at an interval time of 2 min per frame and a total time of 1.5 or 2 h and were exported from Fiji as QuickTime movies to play at 10 frames per sec.

### Post-image Analysis

#### Automated Tracking and Immune Cell-Lesion Behavioural Quantification

All image analysis was performed in Fiji (Schindelin et al., 2012). Detection, tracking and spatial analysis of cells used the Modular Image Analysis (MIA) automated workflow plugin for Fiji (Cross, 2020)^1,2^. The numerical values for the settings were derived empirically and chosen to accurately represent the fluorescence signal.

For neutrophils, their cytoplasm was automatically detected in time-lapse movies using a sequential binarisation and connected-components labelling process (Otsu, 1979; Legland et al., 2016), while for macrophages, where cytoplasmic margin is much less clearly defined, we detected their nuclei and using pixel classification (Arganda-Carreras et al., 2017) followed by binarisation at a fixed probability prior to connected-components labelling. Detected objects were subjected to size-based filters to remove noise.

Cell and nuclear surfaces were automatically outlined and the surface-surface distance between each of the immune cell lineages and pre-neoplastic cells or wounds automatically measured in space and time using Fiji. To distinguish interactions involving direct contacts vs. those immune cells that came in “close” proximity to the cancer or wound lesion, we arbitrarily selected 20 μm as a maximum surface to surface neutrophil-lesion distance, as a “close” interaction, and for macrophages, we considered less than 50 μm nuclear surface-lesion surface (accounting for 30 μm average macrophage radius) to be a “close” interaction (see Figure 1A).

These contacts and close proximity interactions were detected by our bespoke algorithm, and quantified, throughout the period of each movie, to give numbers of immune cell-lesion interactions and their individual and summed/overall duration times.

The number of neutrophils and macrophages in the skin of each larva was automatically quantified from time-lapse movies using the protocol as described above.

Neutrophils and macrophages were tracked between frames using the TrackMate plugin for Fiji (Tinevez et al., 2017). Behavioural features (velocity and directionality ratio) were measured for each tracked cell.

#### Manual Supplementation to Our Automated Tracking Studies

To complement our automated analysis above, the detection of both macrophage nuclei and pre-neoplastic cells was manually corrected in instances where the difference in size and intensity of fluorescence of these objects was not sufficiently great or when cells were overlaying one another.

The margins of the healthy goblet cells or local flank wounds were manually outlined with the Freehand selection tool in Fiji (see Supplementary Figure 1) after visualisation in the red or brightfield channel, respectively, and their dynamic position changes occurring during the movies were iterated by adding multiple manual annotations at different time frames.

Numbers of pre-neoplastic cells and tnfα-expressing macrophages were manually quantified from single confocal images in a pre-defined region/field of view (660 μm × 310 μm) of the flank above the cloaca of each larva.

### Statistical Analysis

Statistical analyses and graph generation were performed using GraphPad Prism 8. Data were confirmed to be normally distributed via D’Agostino-Pearson omnibus or Shapiro-Wilk tests prior to further comparisons. When the data was normally distributed, Student’s unpaired two-tailed *T*-test or ordinary one-way ANOVA with Tukey’s multiple comparison post-test were used to compare two groups or more than two groups, respectively. For non-normally distributed data, Mann-Whitney test or Kruskal-Wallis with Dunn’s multiple comparison post-test were used for comparison between two groups or more than two groups, respectively. Fisher’s exact test was used in the analysis of contingency tables to compare proportions between two groups. In column scatter plots, each dot represents one fish, except for those representing interaction time, velocity or directionality ratio where each small dot represents one cell and larger dots are the mean from one fish. In XY graphs, each dot represents the mean of all fish. In all graphs, which are representative of three independent experiments, the mean is used to calculate the average (horizontal bar), SEM (errors bars) and *p*-value. Statistical significance is indicated on graphs using standard conventions, as follows: n.s., non-significant, *p* > 0.05; ^∗^*p* < 0.05; ^∗∗^*p* < 0.01; ^∗∗∗^*p* < 0.001; ^****^p < 0.0001. The number of fish or cells/fish used in the experiments is indicated for each graph in the figures.

## Data Availability Statement

All datasets presented in this study can be made available upon request. The software used in this study for the analysis of cell behaviour and interactions was developed by SJC and can be found at https://zenodo.org/record/4024567 and https://zenodo.org/record/4700514. Requests to access the datasets should be directed to PL-C, paco.lopezcuevas@bristol.ac.uk.

## Ethics Statement

The animal study was reviewed and approved by the University of Bristol Animal Welfare and Ethical Review Body.

## Author Contributions

PL-C and PM conceived and designed the study, analysed the data, and wrote the manuscript. PL-C carried out all the experiments. PL-C and SJC performed the image analysis. PL-C, SJC, and PM reviewed and edited the manuscript. PM secured the funding and provided project administration, supervision, and resources. All authors contributed to the article and approved the submitted version.

## Conflict of Interest

The authors declare that the research was conducted in the absence of any commercial or financial relationships that could be construed as a potential conflict of interest.

## Footnotes

1 https://zenodo.org/record/4024567

2 https://zenodo.org/record/4700514

## Abbreviations

C.toxins: Coley’s toxins
dpf: days post-fertilisation
dpfi: days post-first injection
dpi: days post-injection
DsRed: *Discosoma* sp. Red
*E. coli*: *Escherichia coli*
GFP: green fluorescent protein
HCs: healthy goblet cells
hpw: hours post-wounding
krt4/19: keratin 4/19
LW: local wound
lyz: lysozyme
mpeg: macrophage expressed gene
nls: nuclear localisation sequence
PNCs: pre-neoplastic cells
RFP: red fluorescent protein
tnfα: tumour necrosis factor α
UAS: upstream activating sequence.
