# Supplementary material for: Modulating the Inflammatory Response to Wounds and Cancer Through Infection
Source: Front Cell Dev Biol. 2021 Apr 30;9:676193. doi: 10.3389/fcell.2021.676193 (PMC8120001; doi:10.3389/fcell.2021.676193)
Supplement: Supplementary file 1 [file Data_Sheet_1.PDF]

Supplementary Material

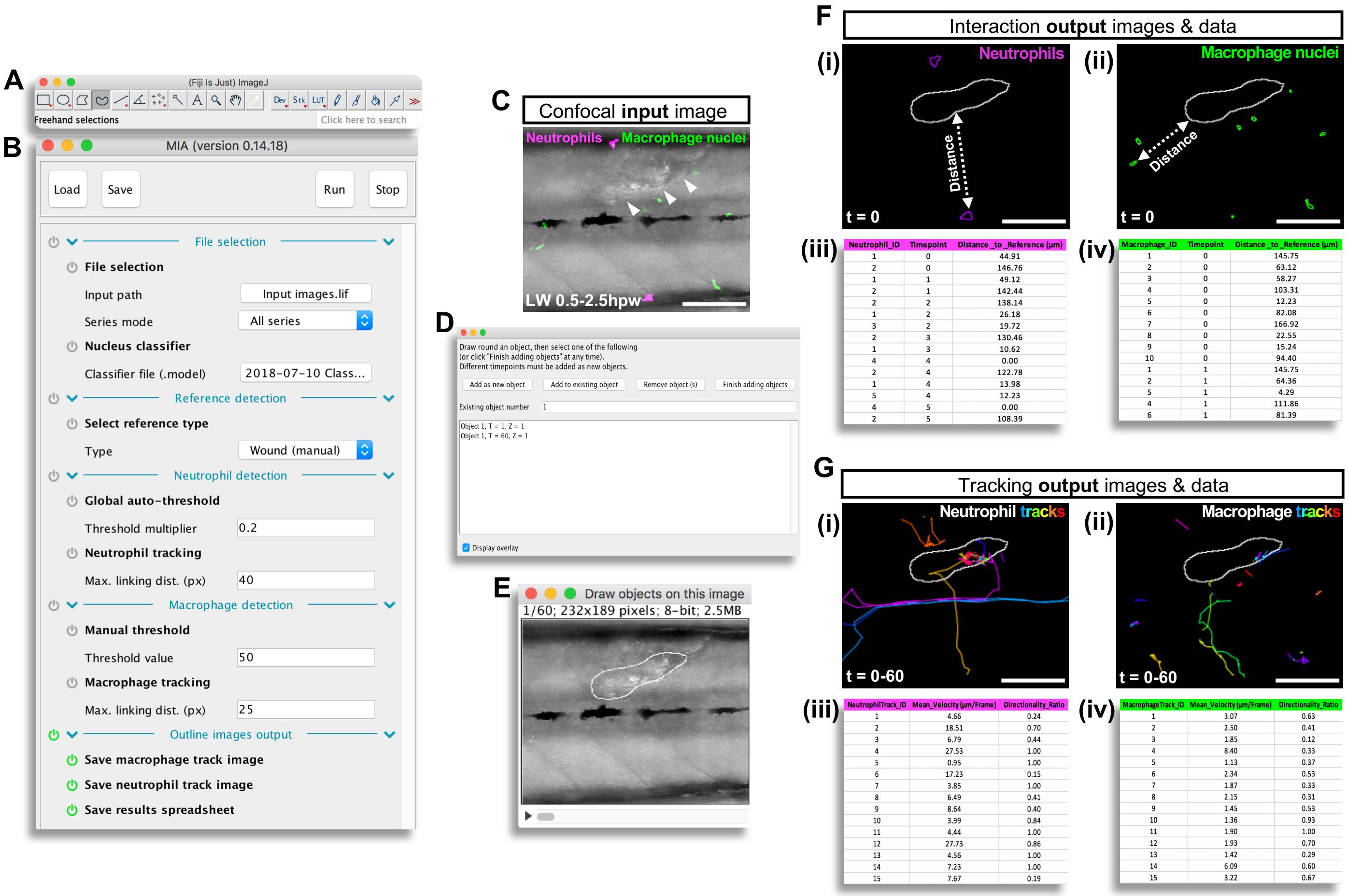

Supplementary Figure S1. Workflow for automated tracking analysis of leukocytes responding to different skin lesions in zebrafish larvae. (A) Fiji toolbar. **(B)** The Modular Image Analysis (MIA) user interface run in Fiji for detection, tracking and spatial analysis of neutrophils, macrophages and skin lesions (termed as reference). **(C)** Multi-channel confocal input image of 6dpf flank wounded Tg(*lyz:DsRed;mpeg1:nls-Clover*) larva at 0.5hpw prior to analysis of neutrophil (magenta) or macrophage (green nuclei) behaviour and their interactions with the wound (white arrowheads). **(D, E)** Drawing tool to manually outline the margins of the wound (white line) visualized in the brightfield channel from (C) at different timepoints (total timepoints=60). Note that the same manual drawing tool is utilised to define the margins of healthy goblet cells, but the pre-neoplastic clone margins are automatically detected. **(F)** Post-software output images from the same larva showing the detection of the margins of neutrophils (magenta) (i), macrophage nuclei (green) (ii) and the wound (white) that is used to calculate the distance of each neutrophil (iii) or macrophage (iv) from the wound (reference) at different timepoints. **(G)** Post-software output images from the same larva showing the neutrophil (i) or macrophage (ii) tracks that are used to calculate both mean velocity and directionality ratio of neutrophils (iii) and macrophages (iv) in the vicinity of the wound. Scale bars=100µm.

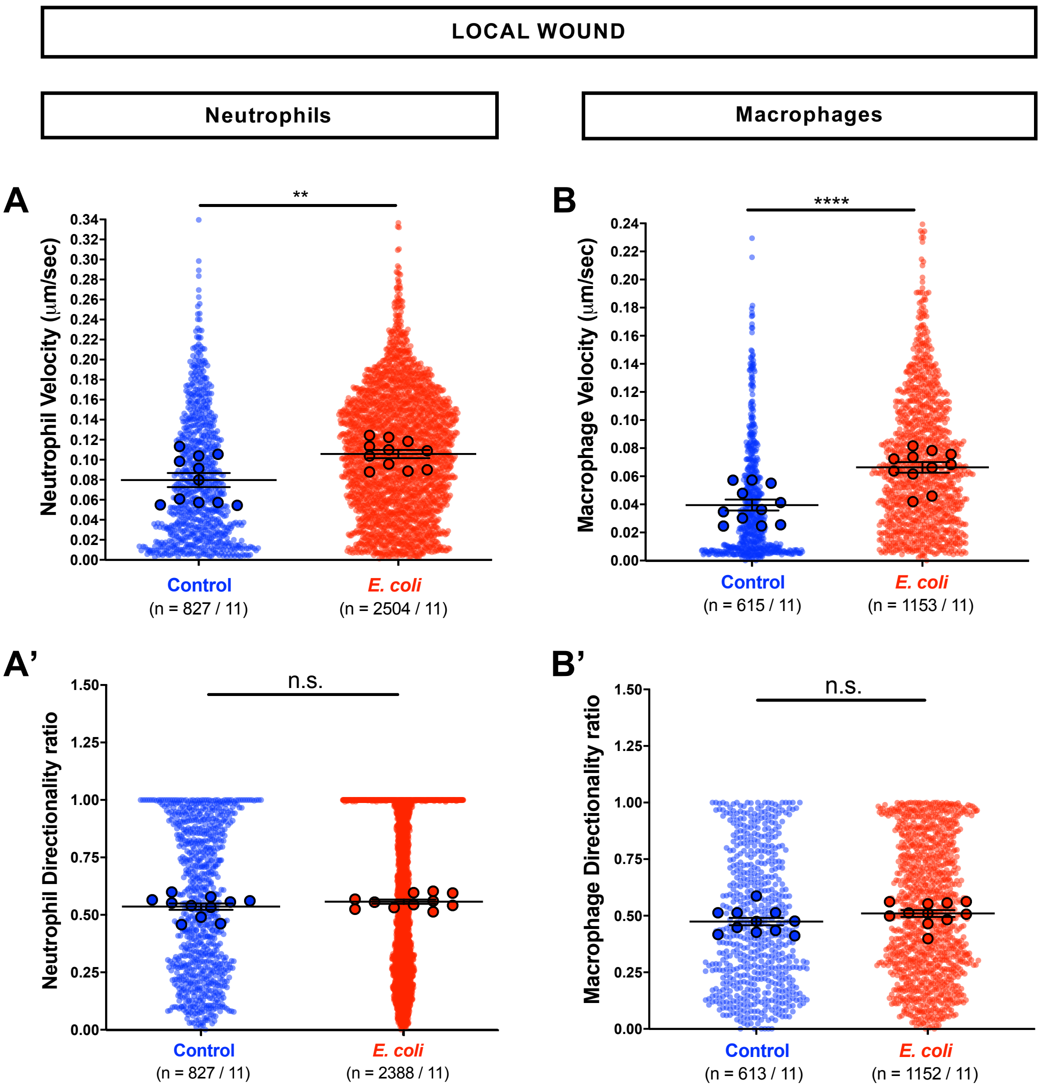

Supplementary Figure S2. Altered behaviour of wound-recruited leukocytes upon *E. coli* infection. (A-D,A'-D') Graphs show velocity and directionality ratio of neutrophils (A,A') and macrophages (B,B') quantified at the wound site from 0.5 to 2.5hpw.

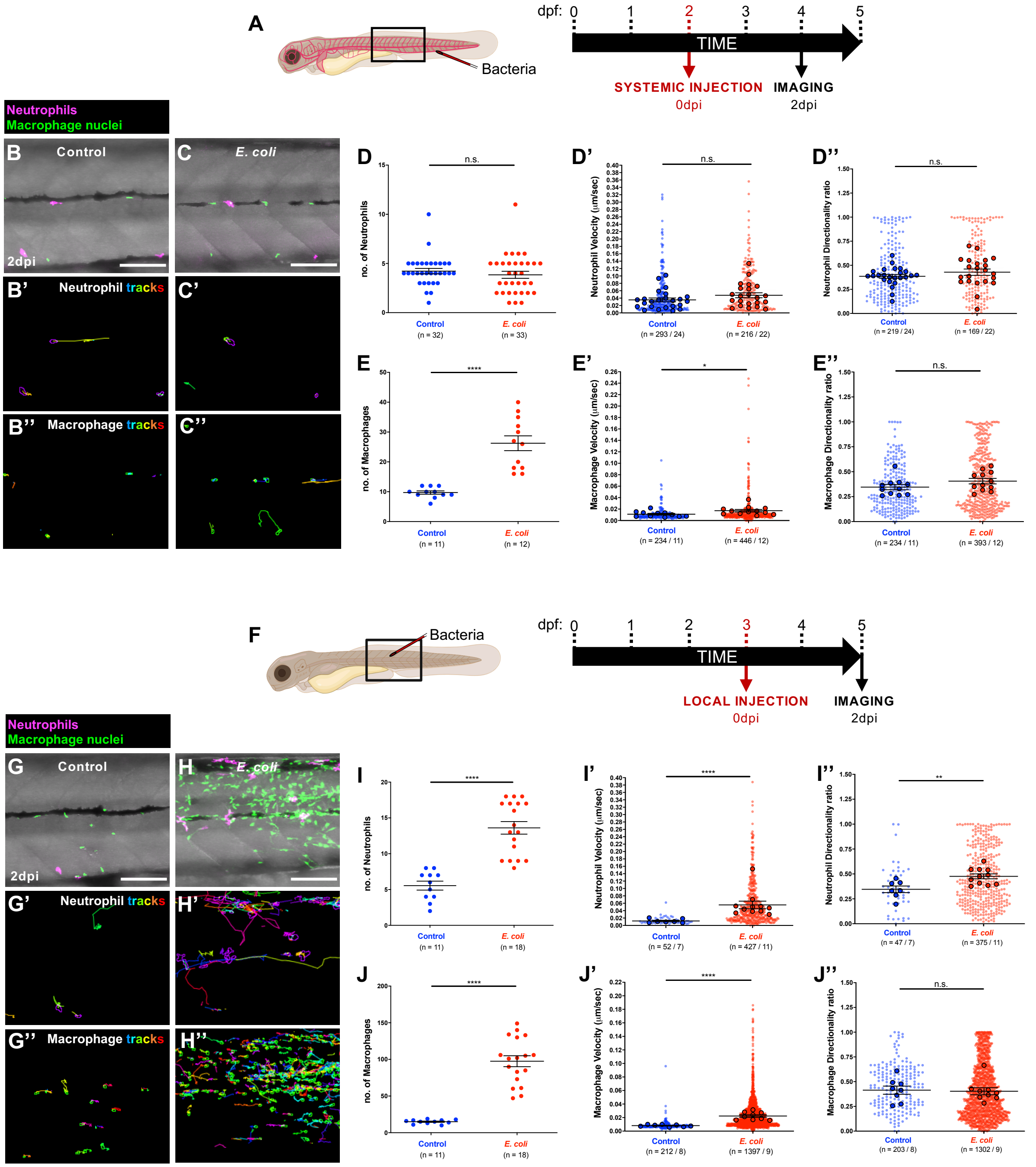

Supplementary Figure S3. Altered numbers and behaviour of leukocytes in healthy zebrafish larval skin upon *E. coli* infection. (A) Schematic of the experimental timeline for systemic infection studies in healthy skin showing the region (black box) to be imaged in (B,C). **(B,C)** Multi-channel confocal movie frames of the flank of Tg(*lyz:DsRed;mpeg1:nls-Clover*) larvae at 2dpi after systemic injection of control media (B) or *E. coli* (C), and prior to analysis of neutrophil (magenta) or macrophage (green nuclei) numbers and their behaviour. **(B',C',B'',C'')** Post-software images of the same larvae showing neutrophils (magenta) (B',C') or macrophage nuclei (green) (B'',C'') and their tracks in the skin. **(D,E,D',E',D'',E'')** Graphs showing number, velocity and directionality ratio of neutrophils (D,D',D'') and macrophages (E,E',E'') quantified in the skin at 2dpi. **(F)** Schematic of the experimental timeline for local infection studies in healthy skin showing the region (black box) to be imaged in (G,H). **(G-J,G'-J',G''-J'')** The same analysis was carried out for local infection as previously described for the systemic infection experiments. Scale bars=100μm.

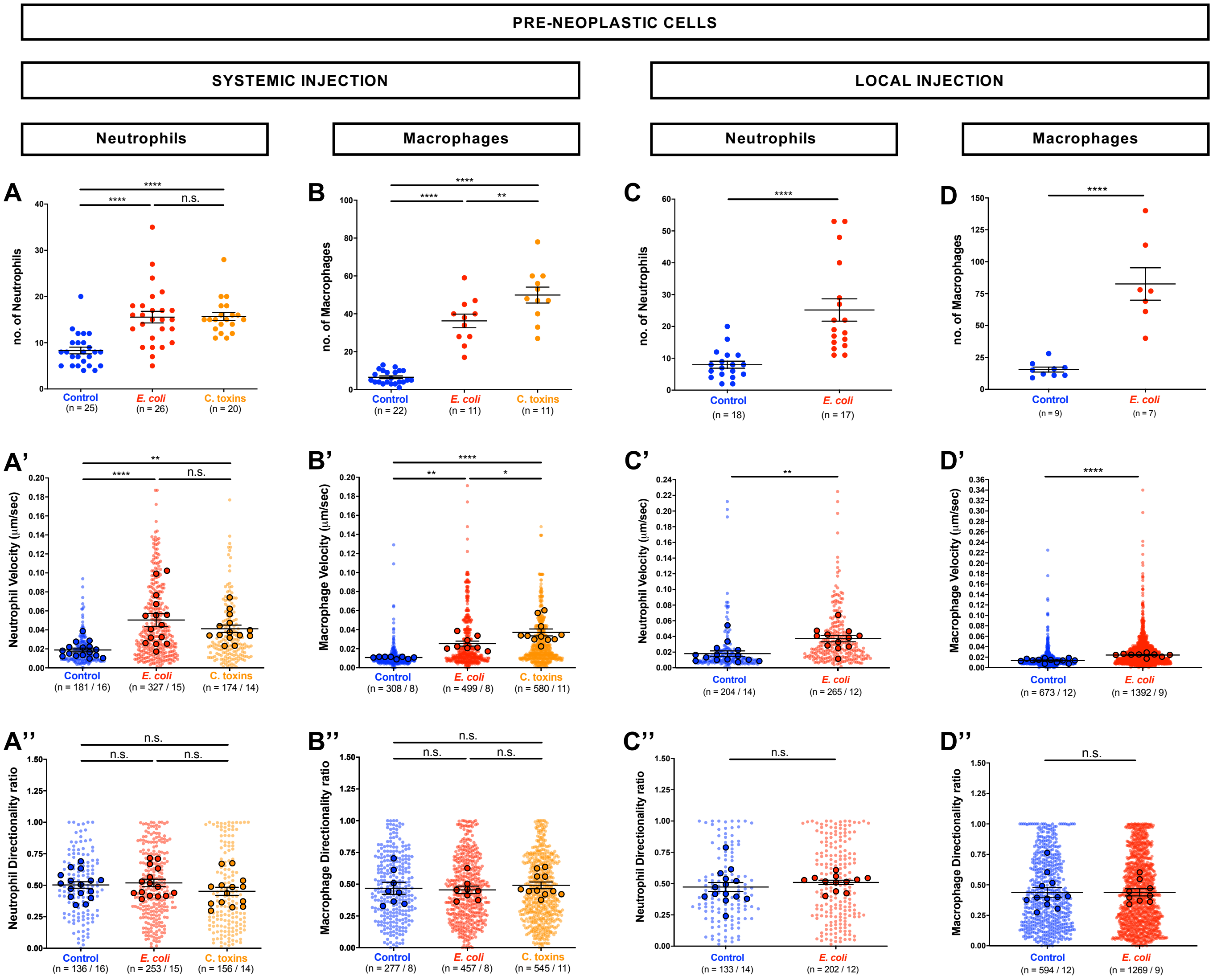

Supplementary Figure S4. Altered numbers and behaviour of leukocytes in the cancerous zebrafish larval skin upon *E. coli* or Coley's toxins infection. (A,B,A',B',A'',B'') Graphs showing number, velocity and directionality ratio of neutrophils (A,A',A'') and macrophages (B,B',B'') quantified in the cancerous larval skin at 2dpi after systemic injection of control media, *E. coli* or Coley's toxins. **(C,D,C',D',C'',D'')** Graphs showing number, velocity and directionality ratio of neutrophils (C,C',C'') and macrophages (D,D',D'') quantified in the cancerous larval skin at 2dpi after local injection of control media or *E. coli*.

	Locally infected wound (vs sterile)	Systemically/locally infected cancer (vs sterile)
**Neutrophil interactions**	-Higher numbers<br>-Higher overall duration	-Higher numbers<br>-Lower individual duration
**Macrophage interactions**	-Higher numbers<br>-Lower individual duration	-Higher numbers<br>-Higher overall duration
**Neutrophil behaviour**	-Increased velocity	-Increased velocity
**Macrophage behaviour**	-Increased velocity	-Increased velocity
**tnfα+ve macrophage numbers**	—	-Higher over time<br>-High initially but declined over time (single infection vs multiple infections)<br>-Majority of macrophages are tnfα+ve over time (multiple infections)
**Wound healing/Cancer cell numbers**	-Delayed wound healing<br>-Majority of wounds still impaired at 24hpw	-Reduced cancer cell numbers (multiple infections vs sterile)

Supplementary Table S1. Table summarising the impact of infection in wound and cancer.

Supplementary Movies

Movie 1. Inflammatory response to a local flank wound, to healthy goblet cells or to pre-neoplastic cells. Time-lapse movies of neutrophils and macrophages interacting with different skin lesions in 6dpf zebrafish larvae.

Movie 2. Inflammatory response to sterile control versus *E. coli*-infected local flank wound. Time-lapse movies of neutrophils and macrophages interacting with flank wounds in 3dpf zebrafish larvae.

Movie 3. Inflammatory response to healthy skin in a systemic control versus *E. coli* infection scenario. Time-lapse movies of neutrophils and macrophages interacting with the healthy skin of systemically injected 4dpf zebrafish larvae.

Movie 4. Inflammatory response to healthy skin in a local control versus *E. coli* infection scenario. Time-lapse movies of neutrophils and macrophages interacting with the healthy skin of locally injected 5dpf zebrafish larvae.

Movie 5. Inflammatory response to pre-neoplastic cells in a systemic control versus *E. coli* infection scenario. Time-lapse movies of neutrophils and macrophages interacting with pre-neoplastic cells in the cancerous skin of systemically injected 4dpf zebrafish larvae.

Movie 6. Inflammatory response to pre-neoplastic cells in a local control versus *E. coli* infection scenario. Time-lapse movies of neutrophils and macrophages interacting with pre-neoplastic cells in the cancerous skin of locally injected 5dpf zebrafish larvae.
